# X-ray tomographic reconstruction and segmentation pipeline for the long-wavelength macromolecular crystallography beamline at Diamond Light Source

**DOI:** 10.1107/S1600577521003453

**Published:** 2021-04-20

**Authors:** Daniil Kazantsev, Ramona Duman, Armin Wagner, Vitaliy Mykhaylyk, Kazimir Wanelik, Mark Basham, Nicola Wadeson

**Affiliations:** a Diamond Light Source Ltd, Diamond House, Harwell Science and Innovation Campus, Fermi Avenue, Didcot OX11 0DE United Kingdom; b Rosalind Franklin Institute, Rosalind Franklin Institute Hub, Harwell Campus, Didcot OX11 0FA, United Kingdom

**Keywords:** long-wavelength X-ray crystallography, absorption correction, segmentation, iterative reconstruction, high-performance computing

## Abstract

In this paper a practical solution for the reconstruction and segmentation of low-contrast X-ray tomographic data of protein crystals from the long-wavelength macromolecular crystallography beamline I23 at Diamond Light Source is provided. The resulting segmented data will provide the path lengths through both diffracting and non-diffracting materials as basis for analytical absorption corrections for X-ray diffraction data taken in the same sample environment ahead of the tomography experiment. X-ray tomography data from protein crystals can be difficult to analyse due to very low or absent contrast between the different materials: the crystal, the sample holder and the surrounding mother liquor. The proposed data processing pipeline consists of two major sequential operations: model-based iterative reconstruction to improve contrast and minimize the influence of noise and artefacts, followed by segmentation. The segmentation aims to partition the reconstructed data into four phases: the crystal, mother liquor, loop and vacuum. In this study three different semi-automated segmentation methods are experimented with by using Gaussian mixture models, geodesic distance thresholding and a novel morphological method, RegionGrow, implemented specifically for the task. The complete reconstruction-segmentation pipeline is integrated into the MPI-based data analysis and reconstruction framework *Savu*, which is used to reduce computation time through parallelization across a computing cluster and makes the developed methods easily accessible.

## Abstract 2

Automatic segmentation methods developed for tomography data collected on macromolecular crystallography beamline I23, Diamond Light Source, to assist with absorption corrections for very long wavelengths X-ray diffraction data are presented.

## 1. Introduction   

Long-wavelength macromolecular crystallography (MX) exploits tender X-rays within a wavelength range of λ = 2–6 Å that covers absorption edges of light atoms of high significance in biology and are natively present in or commonly bound to macromolecules (S, P, K, Ca, Cl). It can be used either for solving the crystallographic phase problem experimentally (Hendrickson, 2000 ▸) or identifying these elements in the resulting electron density maps based on anomalous scattering (Minor *et al.*, 2000 ▸). Anomalous scattering leads to a break in the symmetry of the diffraction pattern from crystals, is element specific with a maximum at the absorption edge, rapidly decreasing on the low energy side and slowly decreasing on the high energy side of the edge.

The macromolecular crystallography beamline I23 at Diamond Light Source is a unique synchrotron instrument dedicated to in-vacuum long-wavelength crystallography experiments (Wagner *et al.*, 2016 ▸). Operating in a high-vacuum environment eliminates air absorption and scattering, resulting in higher signal-to-noise ratios even at the longest wavelengths. However, the impact of absorption by the sample (crystal, sample holder and surrounding mother liquor) is drastic, as photoabsorption is approximately proportional to the cube of the wavelength. While the sample environment and detector allow precise measurement of X-ray diffraction data, in order to deduce accurate structure factors and their anomalous differences from the raw data, an absorption correction needs to be applied. Empirical corrections from the standard software packages [*XDS* (Kabsch, 2010 ▸), *Aimless* (Evans & Murshudov, 2013 ▸)] have been used so far. Unfortunately, for wavelengths longer than 3 Å, the absorption effects are significantly larger, so these corrections are no longer adequate and an analytical correction is needed.

The intensity of X-rays transmitted through a material decreases due to absorption and the extent of these changes varies depending on the size, shape and absorption coefficient of the object under investigation. The premise of analytical absorption corrections for a single-crystal diffraction experiment is well established (Albrecht, 1939 ▸): conceptually it requires the calculation of the transmission factor for all the measured reflections *hkl* (Busing & Levy, 1957 ▸), 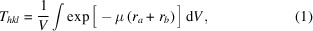
where the integration is over the volume of the crystal *V*, μ is the X-ray absorption coefficient and *r*_*a*,*b*_ are the path lengths of the incident and diffracted beams in the crystal, respectively.

In a typical MX experiment, the X-rays transmit not only through the crystal but also through the sample mount and the surrounding buffer solution. This can be accounted for by expressing the transmission factor as (Santoro *et al.*, 1968 ▸)
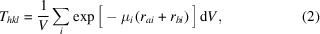
where the summation is performed over *i* materials exposed to the X-rays. This approach relies on the precise knowledge of the geometry of all the objects to determine the path lengths for all measured reflections. This requirement constitutes a major practical complication and it is addressed in this manuscript.

Two methods have been described to overcome this problem. Leal *et al.* (2008 ▸) and Strutz (2011 ▸) have shown that the three-dimensional model of a protein crystal, buffer and sample mount can be constructed using a series of two-dimensional images (silhouettes) taken with an optical sample viewing system (microscope). The resulting model allows the determination of geometrical parameters for absorption correction, but the reconstruction is critically dependent on the image quality commanded by the sample transparency, depth of field, illumination, *etc*. Brockhauser *et al.* (2008 ▸) used for this purpose X-ray microtomography and reported a successful reconstruction of three-dimensional models of protein and DNA crystals including the surrounding solvent and sample holder. This study prompted further developments of tomographic methods to complement and enhance MX, in particular to locate small crystals in lipidic cubic phase (LCP) which is non-transparent at cryogenic temperatures (Warren *et al.*, 2013 ▸; Polikarpov *et al.*, 2019 ▸). X-ray tomography is a promising technique that can aid analytical absorption correction. Recent developments in MX data processing allow very fast feedback to the synchrotron user from automated pipelines (Winter, 2010 ▸). X-ray tomography and the subsequent processing should be linked in these pipelines without or with only minimal user interaction. Automated segmentation is an important aspect of such a pipeline. This was a motivation for including microtomography as a method complementing the long-wavelength MX experiment at beamline I23. The order of the two experiments is important. While the total dose for diffraction data collection is comparable with that required for imaging, radiation damage will affect the higher-resolution information first (Howells *et al.*, 2009 ▸). Therefore, it is necessary to perform the tomography experiment after the diffraction data collection.

In order to provide data required for analytical absorption correction, the 3D volume of the sample needs to be reconstructed from X-ray tomographic projections and segmented for all composite materials (phases). In our case, we need to extract four distinct phases: protein crystal, mother liquor, the loop, and vacuum (background). There are multiple challenges associated with a successful segmentation of X-ray data of protein-based samples. One of the main difficulties is that the chemical composition of protein crystals and their surrounding liquid (mother liquor) are identical, leading to small differences between their linear absorption coefficients and poor absorption contrast between the two phases (Brock­hauser *et al.*, 2008 ▸; Wang *et al.*, 2016 ▸). Additionally, if samples contain various impurities and inclusions, such as protein precipitate from the crystallization buffer or surface of ice crystals, this usually results in irregular intensity distribution, shadowing and streak artefacts in the reconstructed images. Various imaging imperfections can also affect the quality of reconstructions, resulting in ring artefacts and zingers.

In order to overcome these challenges, a robust image processing methodology and efficient software are required. Both image reconstruction and multi-phase segmentation are complex inverse problems meaning that the acquired data are insufficient to determine a unique solution (Bertero & Boccacci, 1998 ▸; Vogel, 2002 ▸). The segmentation result normally depends on the approaches used for tomographic image reconstruction and on the chosen segmentation method. In this study, we endeavoured to solve the general task sequentially by performing two independent steps: an advanced tomographic image reconstruction followed by segmentation of the reconstructed image.

The image reconstruction step is performed by using a model-based iterative reconstruction (MBIR) approach with regularization. With MBIR we aim to enhance contrast, signal-to-noise ratio and suppress image artefacts in the reconstructed images.

In the next step, we segment the reconstructed volume using three different methods: Gaussian Mixture Models (GMM) (Bishop, 2006 ▸; Permuter *et al.*, 2006 ▸), thresholding of the geodesic distance transform of an image (GeoDistance) (Toivanen, 1996 ▸; Criminisi *et al.*, 2008 ▸; Bai & Sapiro, 2009 ▸; Wang *et al.*, 2019 ▸), and a novel region growing (RegionGrow) segmentation technique which performs a set of morphological operations under specific constraints. The results of different segmentation techniques can be combined, providing a multi-phase final segmentation. In order to quantitatively assess the results of the proposed segmentations we also perform manual segmentation for the selected samples.

The paper is organized as follows. In Section 2, we provide information regarding the experimental setup for tomography on beamline I23 at Diamond Light Source (DLS). In Section 3,3 we describe the methodology required for iterative reconstruction, manual and automated segmentations. We also provide details regarding the open-source software. In Section 4, we provide numerical results and quantitative analysis for different segmentation methods. We conclude in Section 5.

## 2. Microtomography setup at I23 beamline   

The main purpose of the I23 beamline is to perform routinely and reliably high-quality MX experiments with long-wavelength X-rays. The solutions chosen to achieve this principal goal dictated a number of unique features adopted by the experimental station (see Wagner *et al.*, 2016 ▸). This inevitably affected construction and operation of all components, apparatus and systems used to either enable the diffraction experiment or enhance it. Two major requirements for any system integrated in the I23 experimental station are (i) compatibility with the vacuum environment and (ii) absence of any interference with the diffraction experiment, which practically means no shadowing of diffracted X-rays. Consequently, the I23 tomography system is designed literally to ‘fit around’ the MX experiment. One compromise is the shared use of the MX multi-axis goniometer with a horizontal rotation axis for both diffraction and X-ray tomography data collections. As a result of variable gravitation pull, such a goniometer has larger centering errors, compared with more stable vertical orientation of the axis implemented at imaging beamlines where all efforts are focused on perfecting the X-ray tomography experiment.

The basic design of our tomography system follows the traditional concept of X-ray imaging with crystal scintillators, shown to be a successful approach for obtaining micrometre spatial resolution (Koch *et al.*, 1998 ▸): X-ray shadow images of a sample are created on a scintillation screen and imaged via microscope optics onto the chip of a CCD camera.

**Figure 1:**
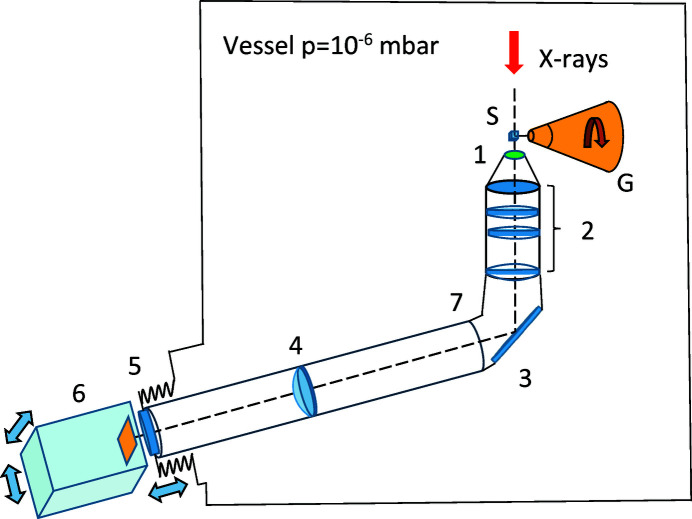
A schematic drawing of the tomography system. 1 – scintillation screen, 2 – objective, 3 – mirror, 4 – relay lens, 5 – vacuum window, 6 – tomography camera, 7 – L-shaped tube. The system is residing inside of a vacuum vessel with pressure bellow 1.0 × 10^−6^ mbar. X-rays are impinging upon a protein sample (S) mounted on the goniometer (G).

In the schematic representation of the system in Fig. 1 ▸ the scintillation screen (1) is a 9 µm film of LSO–Tb grown by liquid phase epitaxy on a 170 µm-thick LSO crystal substrate. The scintillator with the emission peak at 550 nm exhibits enhanced light yield and reduced afterglow, when compared with other analogues (Martin *et al.*, 2009 ▸).

**Figure 2:**
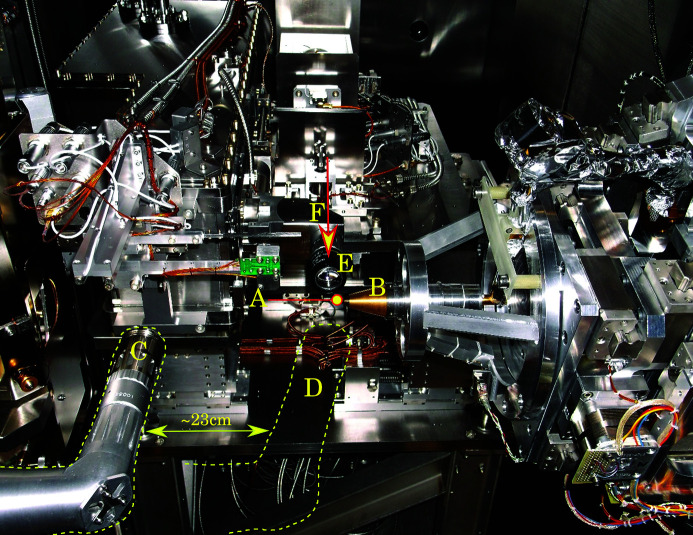
A: Sample position; B: goniometer; C: tomography camera, retracted position; D: tomography camera, in-beam position; E: on-axis viewing system; F: X-ray beam direction.

The light is collected by a 20× Optem objective (2) with numerical aperture NA = 0.42. The optical path is folded by using a mirror (3) and extended by adding a relay lens (4) inside of the L-shaped tube (7). The rationale behind this is to place the CCD camera outside the vacuum vessel. The tube end is welded to the vacuum flange with an optical window (5) which transmits the light to the camera (6). The flange is joined with the vacuum vessel by flexible bellows and mounted on a motorized XYZ stage. This arrangement allows positioning of the tomography system in the beam and a fine adjustment of the scintillator. The air-cooled 14-bit PCO 1600 camera has a 1600 × 1200 pixel CCD with a pixel size of 7.4 mm × 7.4 mm and maximum frame rate of 30 s^−1^. The optical system provides a field of view of diameter 0.6 mm and lateral resolution of ∼1 µm. For diffraction experiments, the assembly is retracted back to the side of the end-station (see C in Fig. 2 ▸). Switching to tomography experiments involves translating the assembly by ∼23 cm to the working position behind the sample (see D in Fig. 2 ▸). The sample-to-scintillator distance can be adjusted between 0.5 and 10 mm by translating the L-shaped tube along the beam path.

The photograph in Fig. 2 ▸ shows the view inside the vacuum vessel from the perspective of the diffraction detector. The tomography system (objective with scintillation screen) is in the retracted position (C), used for collecting diffraction data. The position of the system for collection tomography data is shown with dotted lines (D).

### 2.1. Sample preparation   

Crystals for all three protein samples were prepared by the sitting drop vapour-diffusion technique, at 20°C, in 96-well XtalQuickX crystallization plates. Thaumatin crystals (space group *P*4_1_2_1_2, unit cell 57.5 Å, 57.8 Å, 150.12 Å, 90°, 90°, 90°, solvent content 44%) grew in 0.2 µl + 0.2 µl drops consisting of 50 mg ml^−1^ solution of protein powder (Sigma, T7638) dissolved in deionized water mixed with crystallization solution consisting of 0.050 *M* ADA buffer, pH 6.8, 0.6 *M* potassium sodium tartrate (dissolved in DTNB-saturated water) and 20% glycerol. Thermolysin crystals (space group *P*6_1_22, unit cell 92.7 Å, 92.7 Å, 128.7 Å, 90°, 90°, 120°, solvent content 47%) were prepared by mixing a 25 mg ml^−1^ solution of protein powder (Sigma P1512) dissolved in 0.05 *M* MES buffer, pH 6.0, 45% DMSO and 0.05 *M* sodium chloride with well solution consisting of 1.2 *M* ammonium sulfate, in a 1:1 ratio, using 0.2 µl + 0.2 µl drops. Insulin crystals (space group *I*2_1_3, unit cell 78.18 Å, 78.18 Å, 78.18 Å, 90°, 90°, 90°, solvent content 65%) grew in 0.2 µl + 0.2 µl drops prepared by mixing a 50 mg ml^−1^ solution of protein powder (Sigma I5500) dissolved in 0.01 *M* sodium acetate, pH 3.8 with well solution consisting of 0.1 *M* sodium acetate, pH 4.8, 10% sodium chloride and 25% ethylene glycol.

**Figure 3:**
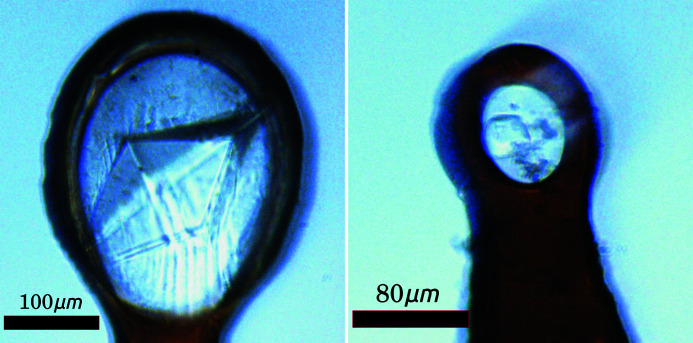
Images of the thaumatin (left) and insulin (right) crystals taken with the on-axis viewing system of the I23 end-station.

Thaumatin and insulin crystals (see Fig. 3 ▸) did not require any cryo-protection prior to vitrification in liquid nitrogen; however, thermolysin crystals were cryo-protected by adding 1 µl of protein buffer to the crystallization drop, immediately before harvesting. Crystals were harvested on dedicated I23 sample holders, compatible with the in-vacuum cryo-cooling requirements of the I23 beamline sample environment. Samples were transferred to the vacuum environment as previously described (Wagner *et al.*, 2016 ▸).

### 2.2. Tomography data collection   

While we experimented with a range of energies for data collection (3–9 keV), the lower energy regime clearly gave better quality data, due to increased absorption of X-rays by matter. The X-ray energies for data collection, 4 keV and 4.5 keV, were chosen as a balance between optimal signal to noise on the detector and exposure time. Data collected at energies outside this range required increased exposure times. Data were collected at an energy of 4.5 keV for thermolysin and 4 keV for thaumatin and insulin, with un-attenuated beam slitted to 0.6 mm × 0.6 mm. The thermolysin dataset consisted of a series of 20 dark images (X-ray shutter closed), followed by 20 flat-field images (sample translated out of the beam using the goniometer X-axis), 1800 projections and 20 flat-field images at the end, taken with a sample to scintillator distance of 1.2 mm. For thaumatin and insulin, data consisted of 40 dark images, 40 flat-field images and 1800 projections, followed by 40 flat-field images, taken with a sample-to-scintillator distance of 0.5 mm. All images were recorded with an exposure time of 0.1 s whilst the sample was rotated with a constant speed over the range of 180° (hardware-triggered on-the-fly scan), with all data being collected within 7 min.

## 3. Methodology and software   

**Figure 4:**
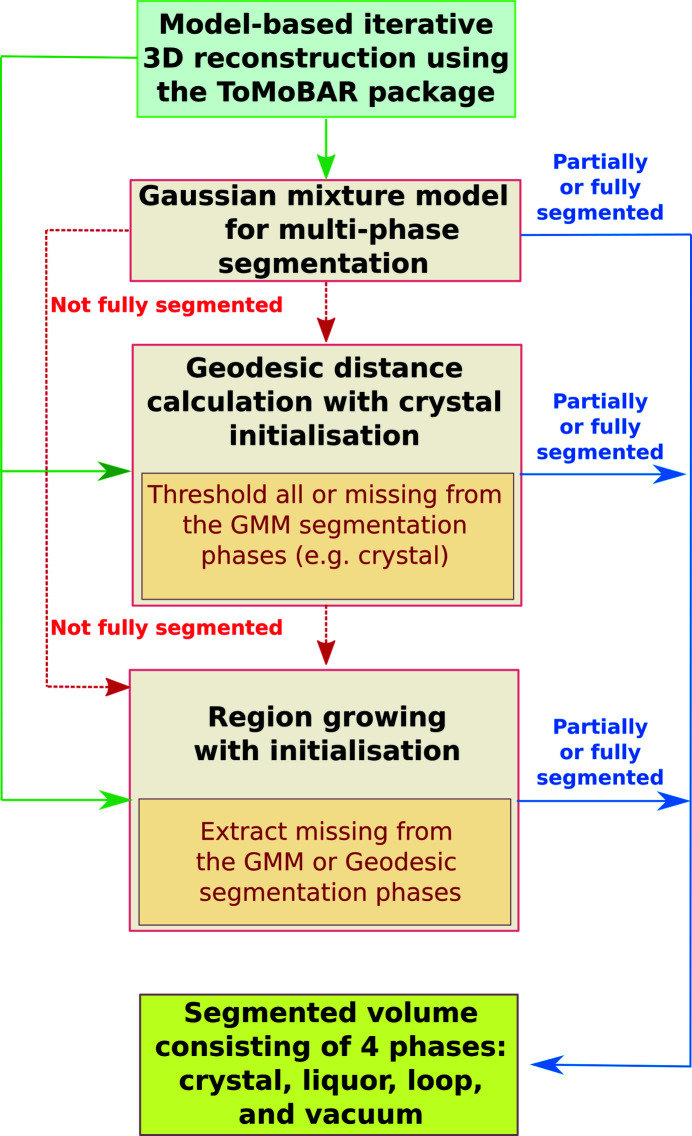
A top-to-bottom data processing pipeline where the first block is a reconstruction with MBIR followed by three complimentary segmentation methods. The whole reconstruction-segmentation pipeline is incorporated into *Savu* software (see Section. 3.3) to ensure fast data access, processing speed and the ease of use.

The overall strategy for segmenting the data consists of two major components: the MBIR of tomographic data followed by segmentation of the reconstructed data into four phases or classes, namely protein crystal, mother liquor, the loop, and vacuum (background). Fig. 4 ▸ shows a block-scheme of the implemented data processing steps, starting from the top block of 3D MBIR and down to multi-phase segmentation using various segmentation methods. Notably, the grayscale reconstructed volume can be passed to three different segmentation methods which can be used independently; however, the final result is usually a combination of multiple segmentation techniques. For instance, the MBIR reconstructed image can be partially segmented using the GMM and then passed to the RegionGrow method to segment the remaining phases (*e.g.* the crystal). Therefore, in this case, the resulting segmented image is a combination of GMM and RegionGrow segmentations.

The reasoning behind this multi-stage segmentation approach is to ensure some flexibility with regard to variations in the data. In our case the data vary significantly depending on the geometry of samples, energy of the beam and the presence of multiple imaging artefacts. In this situation it is hard to establish a single segmentation technique equally suitable for all cases.

In the following sections we briefly explain each of the steps given in Fig. 4 ▸.

### 3.1. Model-based iterative reconstruction   

**Figure 5:**
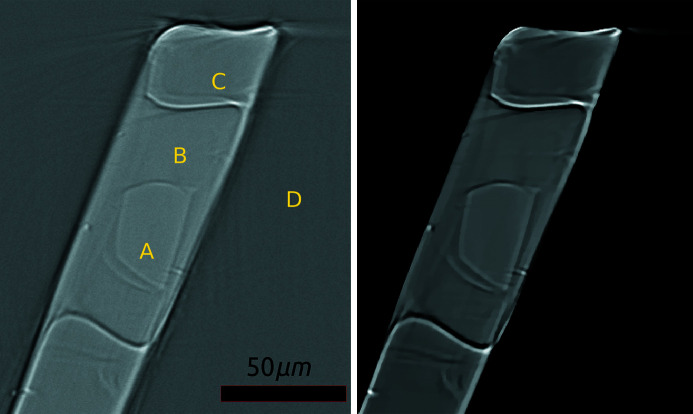
Reconstruction of the insulin sample using the FBP method (left) and iterative reconstruction using *ToMoBAR* (right). The phases are: A: crystal; B: mother liquor; C: loop; D: vacuum.

It is known that the direct reconstruction techniques do not provide the same reconstructed image quality as iterative methods (Bertero & Boccacci, 1998 ▸; Vogel, 2002 ▸). In order to improve image contrast, enhance edges, provide better spatial resolution and minimize image artefacts, we use the MBIR approach to reconstruct tomographic projection data. Due to smaller field of view and access to a computing cluster through *Savu* software, it is feasible to use MBIR routinely. To qualitatively demonstrate the differences in the reconstructed images from the two methods, we present the results from the filtered-backprojection (FBP) direct reconstruction and the MBIR of the insulin sample in Fig. 5 ▸. Notice the significant edge enhancement and noise suppression for MBIR, compared with FBP. Unfortunately, due to imaging conditions and sample characteristics, the boundaries of phases are inconsistent and absorption coefficients of A, B and C phases are very close to each other. Notice also the presence of double-edges (explained in Section 5) and other reconstruction artefacts (streaks and shadowing). The aforementioned factors make the subsequent segmentation a highly challenging task.

In X-ray tomography, the reconstruction task is to recover the unknown attenuation coefficient distribution (the absorption map) 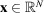 using the log-corrected normalized tomographic projection data 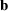 = 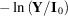, where 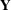 = 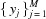 are raw measurements (photon counts) and **I**_0_ is the intensity of the incoming beam.

The following regularized optimization problem needs to be solved, 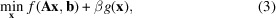
where 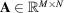 is the system projection matrix, 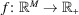 is a continuously differentiable data misfit term, 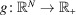 is a convex penalty, and β is the regularization parameter.

For our reconstructions we use the Penalized Weighted Least Squares model for the data misfit, 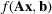 = 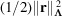, where 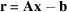, 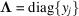, and the Total Variation (TV) penalty (Rudin *et al.*, 1992 ▸) for regularization: *g*(**x**) = ||∇*x*||_TV_. Here, the TV penalty is a suitable choice to ensure a piecewise-constant image recovery due to the presence of sharp geometrical features. The general optimization problem for (3) is solved using A Fast Iterative Shrinkage-Thresholding Algorithm (FISTA) (Beck & Teboulle, 2009 ▸).

We use the *TOmographic MOdel-BAsed Reconstruction* (*ToMoBAR*) software (Kazantsev & Wadeson, 2020 ▸) to reconstruct the projection data. The *ToMoBAR* software uses projection-backprojection operators of the *ASTRA Toolbox* (Van Aarle *et al.*, 2016 ▸) and regularization modules of the CCPi-Regularisation Toolkit (Kazantsev *et al.*, 2019 ▸).

### 3.2. Segmentation techniques   

The segmentation challenge involves partitioning the grayscale image/volume into non-intersecting multiple segments according to some underlying properties. In our case, we consider absorption of four distinct phases: the crystal, the sample holder (loop), mother liquor and vacuum.

The implemented segmentation pipeline consists of three different methods which compliment one another in order to achieve the final result (see Fig. 4 ▸).

#### 3.2.1. Gaussian Mixture Model (GMM)   

The Gaussian mixture clustering process is based on estimating parameters for the *K* number of Gaussian probability density functions (pdfs) given as
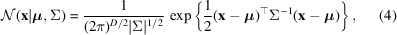
where π is a mixing probability, **x** represents input data points and *D* is the number of dimensions of each data point, **μ** is the mean, and Σ is the covariance matrix.

In order to fit Gaussian pdfs to input data points one can use maximum likelihood estimates to find optimal parameters 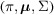. This optimization procedure is implemented in the Scikit–Learn Python library (Permuter *et al.*, 2006 ▸; Pedregosa *et al.*, 2011 ▸). We use it with a fixed number of classes *K* = 4.

The major drawback of this approach (as of any histogram-based segmentation technique) is the dependency on the variations of intensity of different phases. Therefore if the grayscale values of examined phases are close to each other as liquor and crystal (chemically identical) phases in Fig. 5 ▸, this can lead to an ambiguity of overlapping Gaussian pdfs. As a result, mis-classification and phase-merging can occur. Fortunately, some samples (*e.g.* see Section 4.1) can have intensity distinctive phases, so they can be successfully segmented with GMM.

#### 3.2.2. Geodesic distance thresholding (GeoDistance)   

In contrast to the statistical GMM approach, the geodesic distance segmentation is a deterministic technique which also requires some level of user interaction for initialization. It is based on calculating the geodesic distance transform (Toivanen, 1996 ▸; Criminisi *et al.*, 2008 ▸; Bai & Sapiro, 2009 ▸; Wang *et al.*, 2019 ▸) on the reconstructed image and then thresholding the result to obtain the examined phases.

The geodesic distance *D*_geo_ is initiated by user selection in order to classify the pixels belonging to the foreground (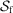) and the background (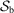). The unsigned geodesic distance for image 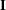 from pixel *i* to the scribble set 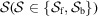 is given as
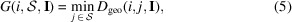


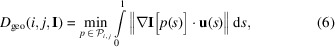
where 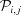 is the set of all paths between pixels *i* and *j*, and *p*(*s*) indicates one feasible path parameterized by *s* ∈ [0, 1]. The unit vector 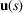 = 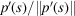 is tangent to the direction of the path.

In order to calculate *D*_geo_ in application to our problem, the user needs to provide a mask which identifies the approximate central area inside the crystal. The mask should be fully enclosed within the crystal area and in theory can be set to any geometrical object, *e.g.* a voxel, a sphere, a cylinder *etc*. We use a curved (pipe-like) cylinder as a mask. After the geodesic distance map is calculated using an initialized mask, the result is thresholded in order to obtain the required phases. The parameters for thresholding can be found manually or automatically.

In this work, we use the implementation of the geodesic distance calculation by Wang *et al.* (2019 ▸) which employs the raster-scan algorithm (Criminisi *et al.*, 2008 ▸). The performance of the GeoDistance method depends on the accuracy of the mask initialized and the subsequent thresholding step. The method can be sensitive to intensity variations inside phases and various image artefacts (*e.g.* ring artefacts, streaks). Conveniently, the initial mask can be provided to the RegionGrow segmentation technique described bellow.

#### 3.2.3. Region growing (RegionGrow)   

This morphological technique is implemented by the authors to enable faster and more controllable 3D segmentation suitable for our purposes. In accordance with other region growing segmentation methods (Pal & Pal, 1993 ▸), the main idea is to compare neighbouring pixels and merge them, if they are similar.

Let 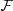 be the set of all voxels in the reconstructed volume 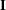, while *P*(…) is a logical uniformity predicate defined on groups of connected voxels. The segmentation problem is to partition 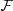 into a set of connected non-intersecting regions 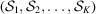 such that
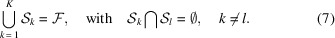
Here 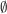 is the null set and 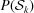 = TRUE for all regions 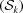 and 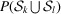 = FALSE for any adjacent regions 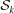 and 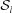.

Let 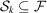 be a user-initialized mask for a chosen phase *k*. First we extract the list of statistical measures associated with the given set 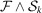: mean (μ_*k*_), mean absolute deviation (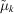), median (ϕ_*k*_) and median absolute deviation (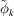). Then the RegionGrow method consists of two main steps:

(1) Iterative expansion of the mask 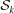 based on the edge detection stopping criteria. If 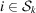 then *j* will assigned to 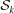 when:

 (i) 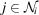 and

 (ii) 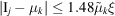 or 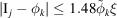.

The iterations are terminated when the conditions above are not fulfilled for any 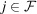.

(2) Iterative removal of morphological noise in 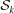. We want to remove gaps, sharp inconsistent features in 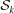 while avoiding erosion of the mask. Therefore, for each 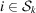 we perform a non-local connectivity search in the neighbourhood of 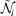.

 (i) To remove gaps if 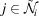 and both 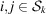, then assign all the voxels on the path between *i* and *j* to 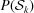 = TRUE.

 (ii) To remove inconsistent features 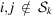 then all voxels on the *i* − *j* path 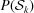 = FALSE.

The *i* − *j* path is calculated using the Bresenham algorithm (Bresenham, 1965 ▸). This discrete line-type of closing is inspired by the work of Soille (2000 ▸). The iterations are terminated when there is no change in 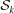 compared with the previous iteration.

The first step of RegionGrow checks the association of voxel *j* to the local neighbourhood 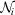 chosen to be 6 (first order) or 26 (second order). The threshold parameter ξ is normally chosen around the value ξ = 1.0 and the final edge value is scaled automatically due to local statistics calculation. In the second step the neighbourhood 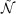 is chosen as 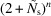, where *n* is the dimension of the input data and 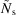 is the size of the non-local searching window.

Although the RegionGrow method is computationally fast, due to parallel implementation, it has several drawbacks. Similarly to the GeoDistance method, it depends on the accuracy of the provided mask. The absence of the closed-curve contour condition can allow RegionGrow evolution in Step (1) to over-segment where the boundary between phases is missing and phases have similar intensity values. The evolution process is also interrupted by the presence of various artefacts resulting in gaps and non-smooth protruding features which should be removed in Step (2). Many iterations of the Step (2) can also lead to over-smoothing of sharp corners; however, this can be minimized by reducing the size of the searching window.

### 3.3. Big data parallel processing with *Savu*   

The challenge of processing big data efficiently is crucial for many synchrotron facilities which can collect up to several petabytes of data annually. At DLS, data are collected from over 30 beamlines and integrated facilities. Therefore, it is crucial to be able to process large amounts of data using parallel computing architectures with the help of the Message Passing Interface (MPI) protocols.

*Savu* is an open source MPI-based tomographic application for large data processing developed at Diamond Light Source (Wadeson & Basham, 2016 ▸). By parallelizing applications across multiple nodes of a computing cluster, one can process a parallel-beam tomographic data significantly faster and more efficiently. *Savu* can be also installed as a standalone application providing quick access to all integrated methods.

The reconstruction-segmentation pipeline presented here has been fully integrated into *Savu*’s framework. This makes it possible to obtain segmented X-ray absorption density maps within reasonable time and minimal user supervision. *Savu*-processed datasets, stored as hdf5 files, can be easily loaded and visualized in another in-house open source data analysis software, *Dawn* (Basham *et al.*, 2015 ▸).

### 3.4. Manual segmentation in *Avizo*   

Manual segmentation for all three datasets was completed using *Avizo Lite* (9.7.0) by labelling four different phases across the stack of the reconstructed images: crystal, solvent, loop and vacuum background. For two of the three datasets, thermolysin and thaumatin, segmentation was started with histogram-based thresholding, using the ‘magic wand’ tool. Segmentation errors caused by reconstruction artefacts were corrected by removing unwanted sections or adding to existing labels using the ‘lasso’ selection tool in ‘auto trace’ mode, followed by interpolation between slices. For the insulin dataset, poor contrast between the crystal and solvent did not allow for histogram-based segmentation; instead, the ‘lasso’ tool and interpolation were used exclusively. Manually segmented data can be used as a golden standard to compare automatic segmentation methods since the ground truth is not accessible. The segmented data were exported as tiff stacks, for further comparison with the automatic segmentation data.

## 4. Numerical results   

Three different protein crystals were chosen for this study, in an attempt to capture different sample morphologies and sizes: thermolysin (a hexagonal rod, approximate size 250 µm × 50 µm × 50 µm), thaumatin (a double-sided prism, approximate size 200 µm × 100 µm × 50 µm) and insulin (a rounded cube, approximate size 20 µm × 20 µm × 20 µm). Manual and automated segmentation results for the three samples are presented in the following sections.

### 4.1. Segmentation of the thermolysin sample   

**Figure 6:**
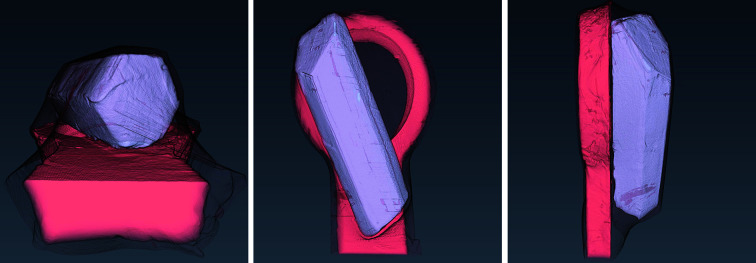
The rendered manual segmentation of the thermolysin sample showing the crystal (light purple), the loop (coral red), and the mother liquor (black mesh).

The appearance of the thermolysin sample is presented in Fig. 6 ▸ via a volume rendering representation of the manually segmented reconstructed data.

**Figure 7:**
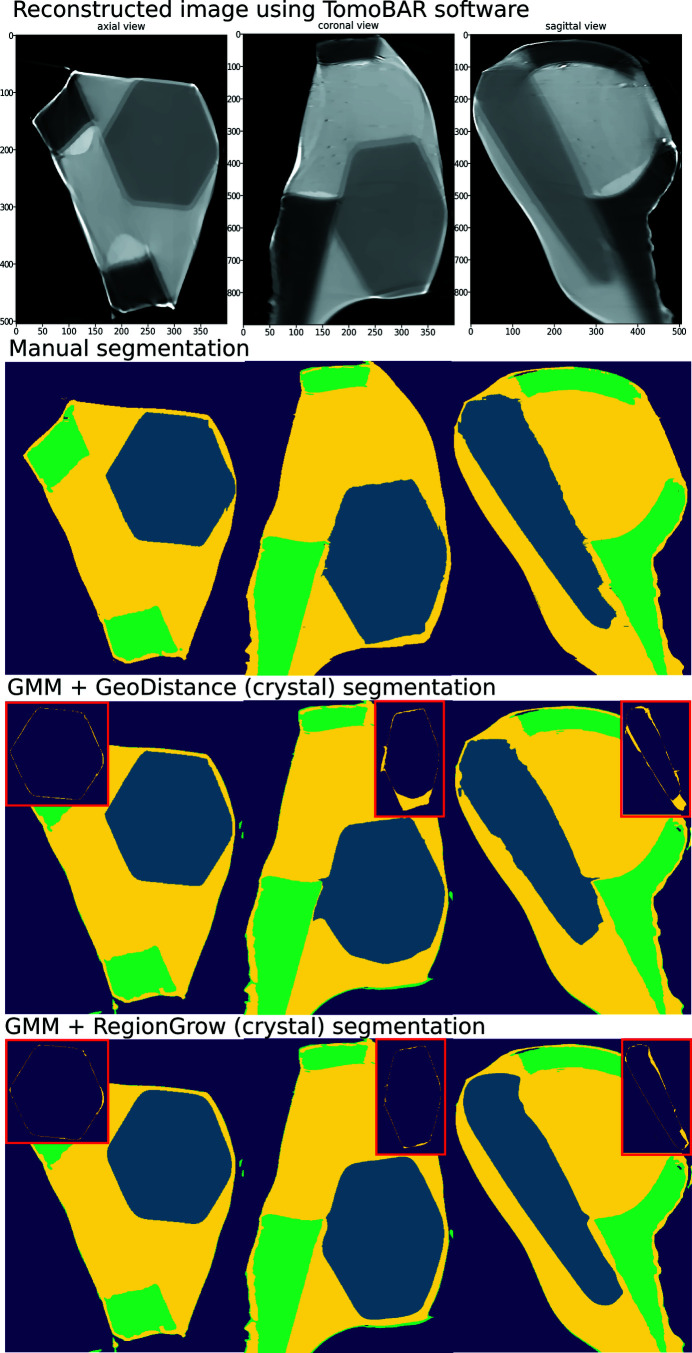
Thermolysin reconstruction and segmentation. Top row to bottom: the result of iterative reconstruction using the *ToMoBAR* package, manual segmentation using *Avizo* (see Section 3.4), a combined segmentation using GMM (liquor, loop and vacuum) and GeoDistance thresholding for crystal, a combined segmentation using GMM and RegionGrow method for crystal. Image discrepancies between the manual segmentation of the crystal and automated methods are presented in the red boxes.

Fig. 7 ▸ (top row to bottom) shows the results of iterative image reconstruction using the *ToMoBAR* package, manual segmentation, GMM segmentation with GeoDistance and RegionGrow for the thermolysin sample. The GMM segmentation is successful in separating the mother liquor, loop and vacuum phases thanks to significant intensity differences between them. The segmentation of the crystal phase is achieved by applying GeoDistance and RegionGrow and then adding to the result of the GMM segmentation. The red boxes highlight the image discrepancies between the manual segmentation and other methods for the crystal phase. These are calculated by taking the absolute difference between the labelled pixels of manual segmentation and automatic segmentation. Notably, the GeoDistance method under-segments the crystal phase while RegionGrow is significantly closer to the manual segmentation, yet slightly smoother. The smoother shape of the crystal with the RegionGrow method is related to the morphological volumetric operation (2) in Section 3.2.3. The more ragged surface of manual segmentation is explained by the extrapolation errors between manually labelled slices.

**Figure 8:**
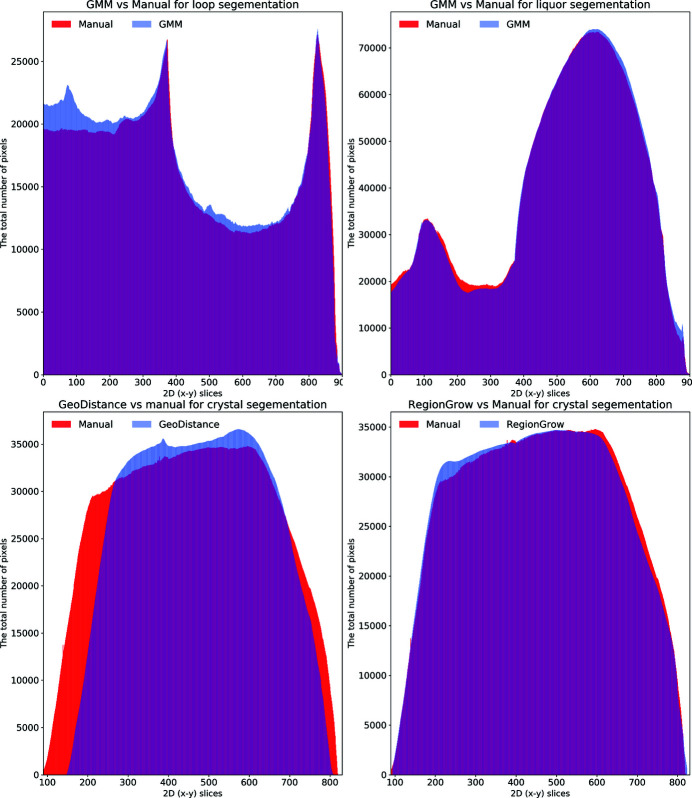
The distribution of segmented pixels of an individual phase with respect to 2D (*x*–*y*) slices for the thermolysin sample. The GMM segmentation for the loop and the crystal is similar to the manual method. For the crystal phase, RegionGrow performs better than Geodistance.

Fig. 8 ▸ shows plots for the total number of segmented pixels for each phase against the 2D (*x*–*y*) slices. These plots are helpful in highlighting how the automated segmentation methods perform compared with the manual one. One can easily identify the over-/under-segmentation in particular regions/slices of the reconstructed volume. There is a very good agreement between the GMM and manual segmentation for the loop and mother liquor phases (top row). The GeoDistance segmentation of the crystal (bottom left) shows a substantial under-segmentation for slices 100–250 and 750–830. Notably, slices 250–750 become over-segmented with the GeoDistance method while RegionGrow (bottom right) performs better and only small parts are mis-segmented.

**Figure 9:**
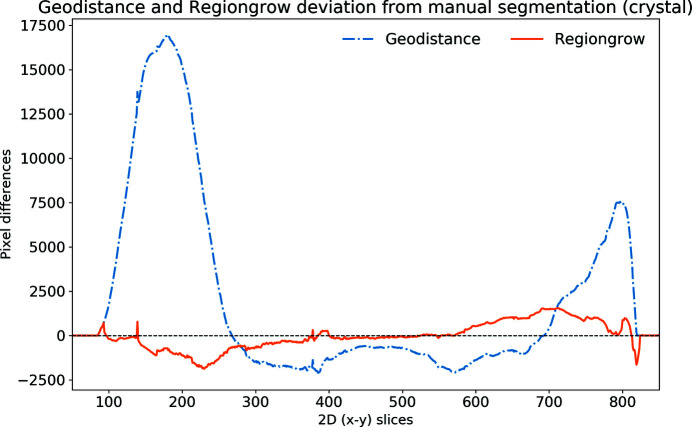
Discrepancy between the automatic segmentation methods GeoDistance and RegionGrow and manual segmentation for the thermolysin crystal. Positive values mean under-segmentation and negative ones over-segmentation. GeoDistance shows a larger deviation from manual segmentation than RegionGrow.

An alternative quantitative representation of the discrepancy between the GeoDistance and RegionGrow segmentations and the manual segmentation, for the crystal phase, is presented in Fig. 9 ▸. It is evident that the GeoDistance method deviates more significantly from the manual segmentation than RegionGrow, especially in the slices corresponding to the edges of the crystal, *i.e.* the first 200 and the last 100 slices in the 2D stack. These are the slices where the crystal is usually elongated with fewer number of pixels in the cross-section. Therefore any errors in segmentation contribute more strongly into quantitative analysis.

### 4.2. Segmentation of the thaumatin sample   

**Figure 10:**
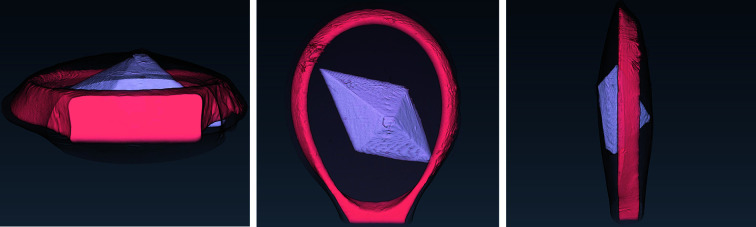
The rendered manual segmentation of the thaumatin sample showing the crystal (light purple), the loop (coral red), and the mother liquor (black mesh).

Volume rendering for the manual segmentation of the thaumatin sample is shown in Fig. 10 ▸.

**Figure 11:**
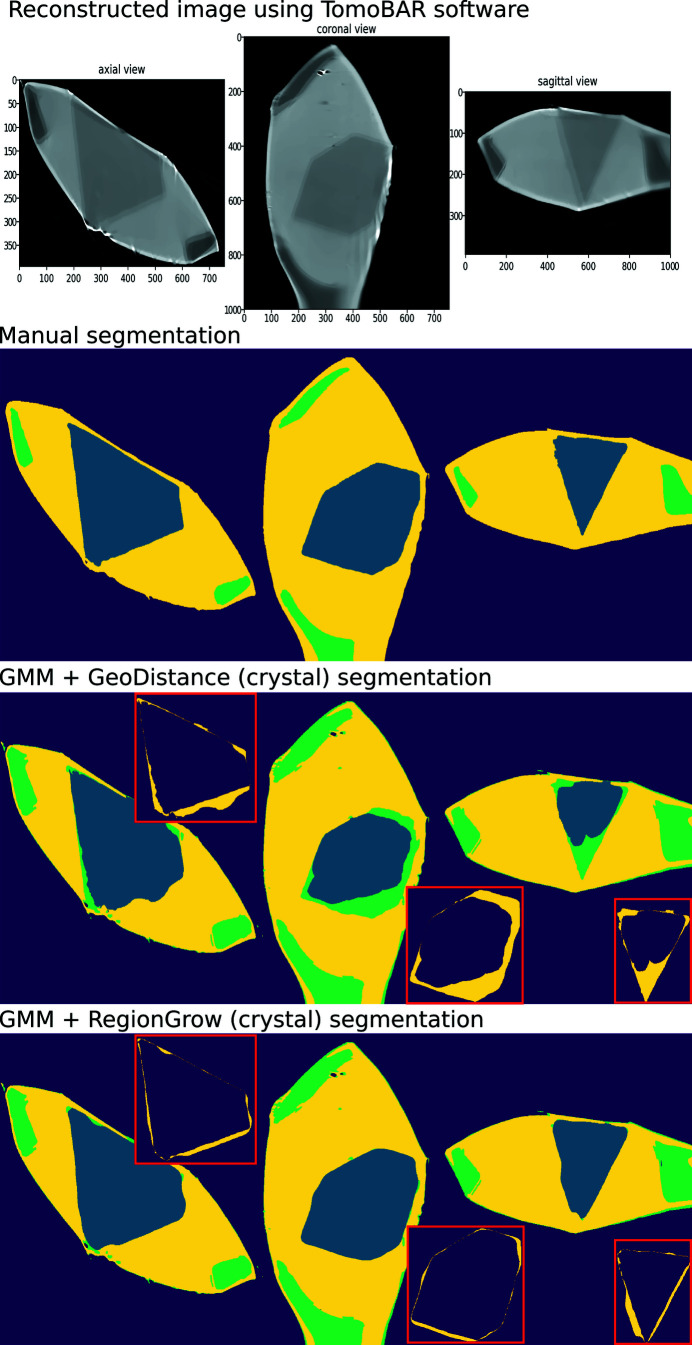
Thaumatin reconstruction and segmentation. From top row to bottom: the result of iterative reconstruction using the *ToMoBAR* package, manual segmentation using *Avizo*, a combined segmentation using GMM (liquor, loop and vacuum), GeoDistance thresholding and RegionGrow for the crystal. One can see that the GeoDistance method under-segments the crystal while RegionGrow is closer to the manual segmentation, yet slightly smoother.

In Fig. 11 ▸, from top row to bottom, we present the results of iterative image reconstruction using the *ToMoBAR* package, manual segmentation, GMM segmentation with GeoDistance and RegionGrow for the thaumatin sample. Similarly to the thermolysin result, the GMM segmentation is successful in segmenting the mother liquor, loop and vacuum phases. GeoDistance and RegionGrow are used to segment the crystal only and the result is added to the GMM result. For this particular sample, GMM erroneously merges the crystal and the loop into one phase. While this is not ideal, the final segmentation result is dictated by the success of the GeoDistance or RegionGrow method in accurately labelling the crystal phase. In this instance, the GMM mis-segmentation is rectified by the result of RegionGrow.

**Figure 12:**
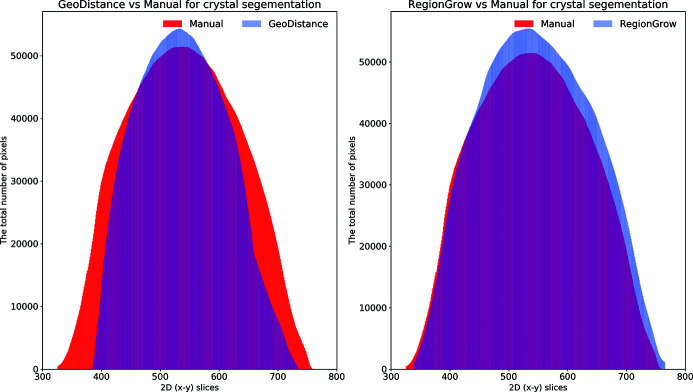
The distribution of segmented pixels of the crystal phase with respect to 2D (*x*–*y*) slices of the thaumatin sample using GeoDistance and RegionGrow methods. It can be seen that the GeoDistance segmentation significantly under-segments, while RegionGrow slightly over-segments.

**Figure 13:**
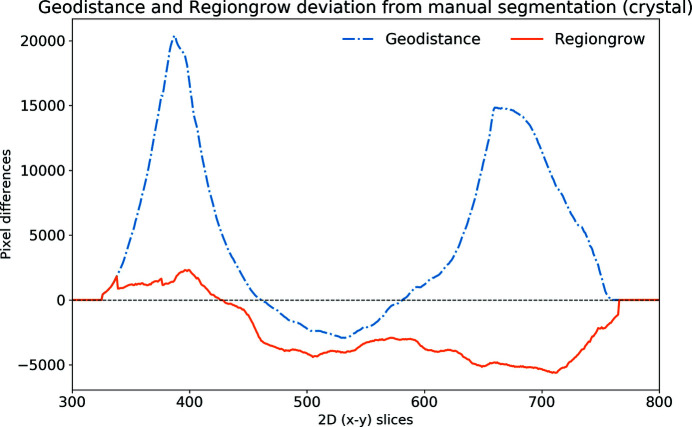
The discrepancy of GeoDistance and RegionGrow segmentation from the manual segmentation for the thaumatin crystal. Note that the deviation of GeoDistance is larger than for RegionGrow. Positive values mean under-segmentation and negative over-segmentation.

The automatic segmentation of the thaumatin sample results in a more significant deviation from the manual method for GeoDistance compared with RegionGrow with respect to the crystal phase segmentation, which is consistent with the result of the thermolysin sample. While RegionGrow is closer to manual segmentation, GeoDistance undersegments the crystal phase as illustrated in Fig. 12 ▸. The segmentation difference plot in Fig. 13 ▸ confirms the qualitative assessment of Fig. 11 ▸ and, again, shows a significant departure from the manual segmentation especially in the regions of the 2D stack corresponding to the edges of the crystal.

### 4.3. Segmentation of the insulin sample   

**Figure 14:**
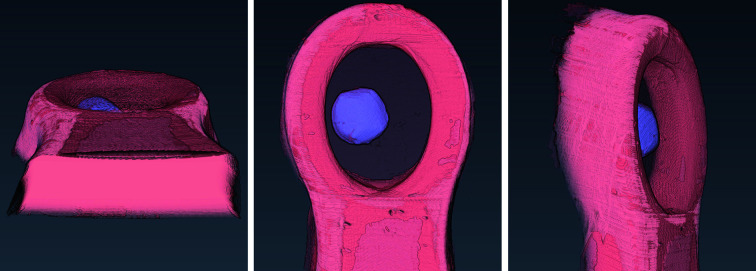
The rendered manual segmentation of the insulin sample showing the crystal (purple) and the loop (red). Mother liquor is not visible in this case.

The appearance of the insulin sample is presented in the volume rendering of the manually segmented data in Fig. 14 ▸. The mounting of this sample resulted in only a thin film of mother liquor surrounding the crystal, which explains why this phase is barely visible in the rendering.

**Figure 15:**
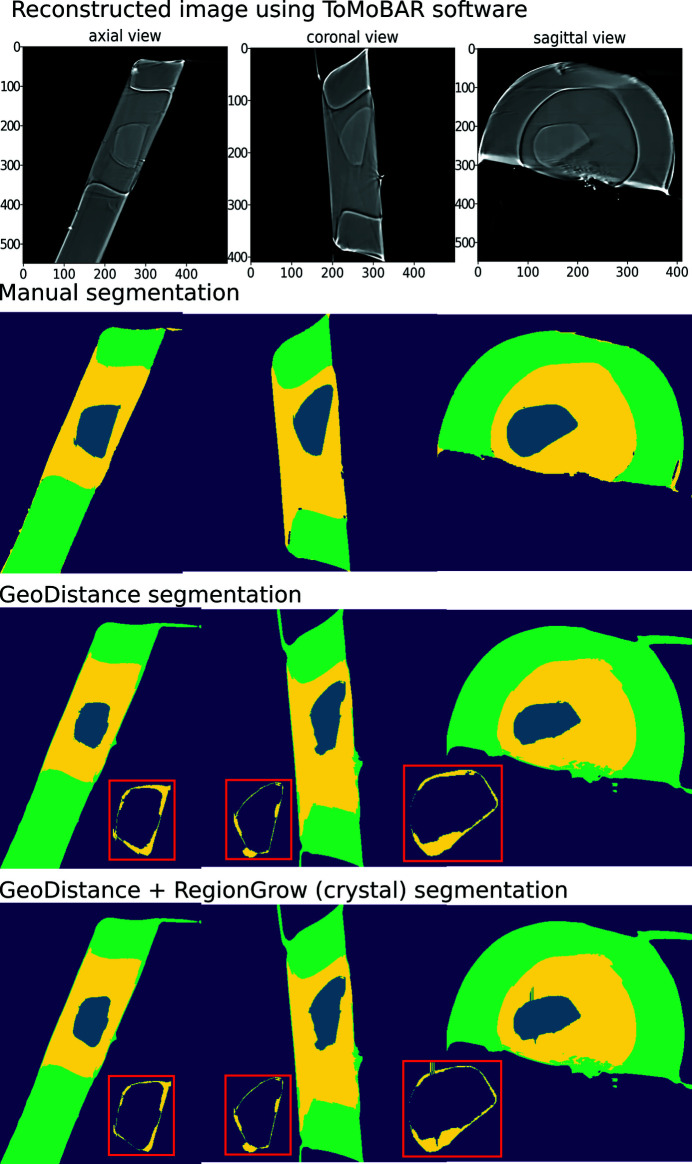
Insulin reconstruction and segmentation. From top row to bottom: the result of iterative reconstruction using the *ToMoBAR* package, manual segmentation using *Avizo*, a complete GeoDistance thresholding segmentation for all phases, RegionGrow segmentation using an input from GeoDistance to segment the crystal. One can see that the GeoDistance method provides a reasonably good segmentation for all phases. RegionGrow adds a small improvement to the existing GeoDistance segmentation for the crystal.

Unlike the other two samples, the insulin reconstruction data offers no contrast between the crystal, mother liquor and loop phases, as shown in the reconstruction results presented in the first row of Fig. 15 ▸. This renders the GMM segmentation method ineffective and, therefore, the only option is to apply the GeoDistance and RegionGrow methods.

The GeoDistance segmentation results, presented in Fig. 15 ▸, third row from the top, show accurate identification of all four phases. As evident from the image errors, the edges of the crystal pose a significant challenge for the automatic segmentation methods. The streak artefacts additionally contribute to the discrepancies between the manual and automated segmentations.

**Figure 16:**
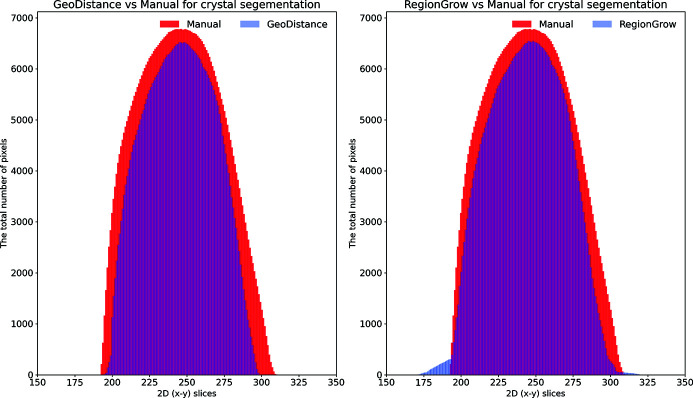
The plots demonstrate the distribution of segmented pixels of the crystal phase with respect to 2D (*x*–*y*) slices of the insulin sample using GeoDistance and RegionGrow methods. The GeoDistance segmentation significantly under-segments while RegionGrow slightly over-segments.

As a quantitative comparison between the two automatic segmentation methods and the manual segmentation, Fig. 16 ▸ indicates that both GeoDistance and RegionGrow under-segment the crystal, confirming the image errors in Fig. 15 ▸. The quality of the reconstruction for the insulin sample is noticeably lower, compared with the other two samples, making segmentation particularly challenging. The presence of multiple artefacts and inconsistent boundaries between phases lead to slightly higher segmentation inaccuracies than expected. Given the segmentation challenge presented by this sample, we take a different processing approach and attempt a combination between the results of GeoDistance and RegionGrow. With respect to the crystal phase, adding the RegionGrow method slightly reduces the discrepancy between the automated and manual methods, by expanding the result of the under-segmented GeoDistance segmentation. Nevertheless, it over-segments in some areas, such as slices 170–190 in Fig. 16 ▸ (right). However this is due to the presence of streak artefacts, so, with better data, this should not be a problem.

**Figure 17:**
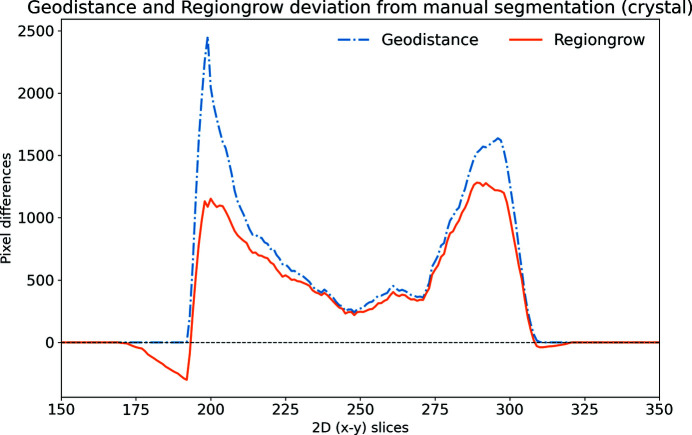
The discrepancy between the GeoDistance and RegionGrow segmentations and the manual segmentation for the insulin crystal. Note that the RegionGrow slightly improves GeoDistance segmentation but mis-segments 170–190 region. Positive values mean under-segmentation and negative over-segmentation.

The segmentation discrepancy between the GeoDistance, RegionGrow and the manual methods is shown in Fig. 17 ▸. Notably, the RegionGrow method performs slightly better than GeoDistance but mis-segments a small region.

For all three samples, the shape of the discrepancy curves (Figs. 9 ▸, 13 ▸, 17 ▸) shows larger disagreements between automatic and manual methods in the region of data corresponding to the edges of the crystal, where the crystal phase represents a small proportion of the entire object and also the presence of artefacts is more pronounced.

## 5. Discussion and conclusion   

In this study we present three complimentary semi-automatic methods for segmenting low-contrast tomographic data collected on the long-wavelength beamline I23 at DLS. These can be used as part of a diffraction and tomography data processing pipeline to account for the significant absorption of very long wavelengths X-rays.

The three different samples in this study were chosen to provide distinct morphologies and sizes for testing the automatic segmentation methods. Our results show that the GMM method is a convenient tool for segmenting some prominent phases, *e.g.* vacuum, loop and mother liquor (see Sections 4.1 and 4.2), when the intensity differences allow it. The result of GMM can then be superimposed with the GeoDistance and/or RegionGrow segmentations of the remaining phases, *e.g.* the crystal phase. If the intensity differences between phases are negligible (see Section 4.3) and GMM fails, as in the case with the insulin sample in our study, GeoDistance can perform segmentation for all required phases; however, it requires more supervised effort. In order to obtain all the phases after the result of GeoDistance calculation, one needs to apply thresholding. This can be done manually, as in this paper, or automatically. The developed RegionGrow method (see Section 3.2.3) performs overall well if the threshold parameter ξ is chosen correctly. Parameter ξ controls the edge detection and, when chosen incorrectly, can lead to over or under segmentation. Additionally, for both GeoDistance and RegionGrow methods, the selection of the mask defines the final result. To avoid mis-segmentation, the mask needs to be fully within the selected phase and must not include any other phases and edges.

All three datasets presented in this study show a feature (double edges at boundaries between different materials) with both positive and negative effects on segmentation and which is an unavoidable effect of the data collection parameters, as well as the nature of samples under investigation. The strong double edges seen in the reconstructed images can be regarded as helpful in providing a boundary between two different phases, while being a hindrance in accurately identifying the physical limit of phases. Their presence can be explained in terms of the interaction of long-wavelength X-rays with low-contrast biological samples.

As protein crystals grow in a liquid medium with identical chemical composition, the difference between the linear absorption coefficients of crystal and surrounding liquid is very small, leading to poor absorption contrast between the two phases in tomographic reconstruction, which severely impedes distinguishing between them for segmentation purposes. Nevertheless, absorption is not the only consequence of interactions of X-rays with matter, as X-rays passing through a sample also undergo refraction. Both absorption and refraction effects are more pronounced at lower energies (*E*), as the two components of the complex refractive index *n* (*n* = 1 − δ + *i*β), the absorption index β, and the refractive index decrement δ, vary with ‘1/*E* ^4^’ and ‘1/*E* ^2^’, respectively. Refraction of X-rays leads to phase changes between the incoming and outgoing radiation, creating phase-contrast effects which cause edge enhancement at the boundaries between materials (Snigirev *et al.*, 1995 ▸). This effect is manifested as visible fringes in tomographic projections which result in the double-edge features seen in our reconstruction images, at the boundaries between crystal and surrounding liquid, liquid and loop, as well as loop and background (see Fig. 5 ▸). Although the edge enhancement is a useful feature for both manual and automatic segmentation, the double-edge creates an uncertainty on the position of the true edge.

While there are some discrepancies between the manual and the automatic segmentation results, the two methods produce comparable data. For difficult cases, like the insulin sample in this study, manual adjustment of the automatically segmented data would be needed to achieve the required results. However, for conventional data, the automatic segmentation offers a significant advantage in saving time. Manual segmentation can take several hours, even up to a day, per dataset, depending on the quality of the reconstructed data and the presence of artefacts.

The presented segmentation methods provide a starting point for developing more robust and automated segmentation techniques. For example, with the fast development of deep learning (DL) classification and segmentation approaches (Minaee *et al.*, 2020 ▸), our next step will be to explore the possibility of fully automated segmentation methods. Further improvement of the presented segmentations can be obtained with an improvement of the reconstruction stage. The model-based reconstruction can be better tuned to minimize various imaging artefacts. Equally, state-of-the-art DL denoising algorithms can do a better job of removing noise than traditional regularization methods.

These improvements will allow us to focus on the next important steps towards absorption corrections for long-wavelength crystallography, such as the quantitative description of the segmented volumes and calculation of their corresponding absorption coefficients.

In this paper, we demonstrated a multi-component pipeline including model-based reconstruction and segmentation of tomographic data. The pipeline has been successfully integrated into the higher-level MPI-based software *Savu* to provide fast access to data and computing resources at DLS. These results represent an important and promising step towards the development of a combined diffraction and tomography pipeline aimed at providing absorption-corrected long-wavelength data and thus enabling the unique experimental opportunities that come with using the longest wavelengths beamline I23 can access.
